# The Greatest Challenge to Using AI/ML for Primary Health Care: Mindset or Datasets?

**DOI:** 10.3389/frai.2020.00053

**Published:** 2020-08-21

**Authors:** Erica L. Troncoso

**Affiliations:** Technical Leadership and Innovations, Jhpiego, Johns Hopkins University, Baltimore, MD, United States

**Keywords:** artificial intelligence, machine learning, primary health care, low and middle income countries, health system, global health, health data, precision public health

## Abstract

The global vision for primary health care (PHC) is defined by regular access to quality care for comprehensive services throughout the course of life. However, this is not what typically happens, especially in low- and middle-income countries, where many people access the formal health system only for emergent needs. Yet, even episodic care is nearly impossible to attain due to infrastructure barriers, critical shortages of health care providers, and low-quality care. Artificial intelligence and machine learning (AI/ML) can help us revolutionize the current reality of health care into the vision of continuous health care that promotes individuals to maintain a constant healthy state. AI/ML can deliver precise recommendations to the individual, transforming patients from a passive receiver of health services into an active participant of their own care. By accounting for each individual, AI/ML can also ensure equitable coverage for entire populations with an ongoing data exchange between personal health, genomic data, public health, and environmental factors. The greatest challenge to enlisting AI/ML in the quest toward the PHC vision will be instilling a sense of responsibility with global citizens to recognize health data for the global good while prioritizing protected, individually owned data sets. Only when individuals start taking a collective approach to health data, shifting the mindset toward the goal of prevention, will the potential of AI/ML for PHC be realized. Until we overcome this challenge, the paradigm shift of the global community away from our *ad hoc*, reactive health system culture will not be achieved.

The global vision for primary health care (PHC) is defined by regular access to quality care for comprehensive services throughout the course of life. The World Health Organization (WHO) states that it has three established components: (1) meeting people's health needs throughout their lives; (2) addressing the broader determinants of health through multisectoral policy and action; and (3) empowering individuals, families, and communities to take charge of their own health (World Health Organization and the United Nations Children's Fund (UNICEF), 2018). Importantly, PHC emphasizes well-being, people-centered care rather than disease-centered care, by providing care through the community. However, this is not what typically happens, especially in low- and middle-income countries (LMICs), where many people access the formal health system only for emergent needs (Barocas and Selbst, 2016). The concept of “health care” from this perspective is something that is sought from a person at a single point in that individual's life when the need arises, allowing the episodic nature to flourish. Yet even episodic care is nearly impossible to attain in LMICs due to the missing pieces from the broader determinants of health: infrastructure barriers, critical shortages of health care providers, and low-quality care (Bitton et al., 2017). While the current state of most LMIC health systems remains weak and fragmented, those in the most disadvantaged and remote populations will remain, as always, at highest risk for being left behind without health care coverage or having access only to poor-quality care. This demonstrates the shocking distance that most health systems in LMICs are away from the PHC paradigm. Though the global health community has committed to operationalize PHC through the 2018 Astana Declaration, health systems in LMICs are struggling due to existing tensions between different vertical and horizontal approaches, non-integrated systems, and poor coordination among all actors of the health system (Bitton et al., 2017; Celi et al., 2019).

More than ever, global health organizations, national governments, foundations, and large non-profits and for-profits have released resolutions, announced digital health strategies, and focused financial efforts all around harnessing the power of digital health and artificial intelligence as a critical component to advancing universal health coverage (UHC) and the health aims of the Sustainable Development Goals (SDGs) (Dressel and Farid, 2018; Druetz, 2018; Floridi et al., 2018; Flahault et al., 2020). The WHO advocates, in their PHC Operational Framework (Frenk, 2009), for the “use of modern health information and communication technology (ICT) in ways that improve effectiveness and efficiency and promote accountability” and acknowledges that “it is impossible to deliver PHC effectively and efficiently without accurate, up-to-date data about the health status of individuals and the population and the current state of the health system.” However, digitalization of health care is accelerating without accounting for the current paradigm of PHC. The current ability of artificial intelligence and machine learning (AI/ML) has matured exponentially, now able to process unprecedented amounts of data to assist with classification for diagnosis, prediction, and optimization for precision treatment (Druetz, 2018; Hosny and Aerts, 2019). With these exciting abilities spotlighting a multitude of potential benefits, there is a risk of AI/ML steering the changes to health systems worldwide, leaving the PHC paradigm behind.

Risk also exists in overlooking or minimalizing the shortcomings that occur today in the health system. Other sectors with flawed systems, such as education, judicial, and labor, have learned the hard lesson of applying algorithms without fixing systemic issues first, now dealing with the resulting detrimental outcomes (Coates et al., 2019). In those systems, algorithms exposed the existing weaknesses and exponentially exacerbated the negative effects, wreaking havoc on already marginalized and disadvantaged lives, such as reproducing a biased and prejudiced justice system and discriminatory practices around hiring (Khoury et al., 2016; Mackey and Nayyar, 2017). Judicial systems using algorithms aimed to predict recidivism have incorporated aspects of data that may be correlated to race in the United States, potentially leading to racial disparities in predictions (O'Neil, 2016). In the labor example, rather than adjusting for the known discriminatory employment and hiring practices, companies hungry for a quick fix unintentionally used discriminatory algorithms to weed out potential candidates from large applicant pools (Khoury et al., 2016). AI/ML is a powerful tool, but that is all it is, and if we apply it to our system of health care now, we will only reproduce the *ad hoc* and reactive pervasive health system culture at a damaging scale. This is where PHC should lead AI/ML work and promote resolving the flaws in the system, before applying algorithms that drive decisions sans human intervention. Like other domain failures, unsupervised decision automation within health care will be a risk to those who may already be marginalized and vulnerable; yet a human of the future augmented by AI/ML to supplement their decision making will surely outpace those without. If all data scientists working within health care were also required to take an oath to do no harm, would this change the way data are assessed and models developed?

The power of AI/ML for PHC will be in its ability to respond to the preventative side of health care, using it as a defensive shield instead of a perfect reactive weapon. However, we need to assess our current health systems and data for the biases, exclusions, inequities, and discriminations now (Khoury et al., 2016). If we can evaluate and identify gaps within our current system, AI/ML can help us revolutionize the current reality of health care into a system that allows individuals to maintain a constant healthy state and meets people's health needs throughout their lives. AI/ML can deliver precise recommendations to the individual, transforming the role of patients from a passive receiver of health services into an active participant of their own care. By accounting for each individual, AI/ML can also ensure equitable coverage for entire populations with an ongoing data exchange between personal health, genomic data, public health, and environmental factors. AI/ML can allow for widespread precision in health care, from precision medicine for the individual to precision public health for populations (Ribeiro et al., 2016; Özdemir and Springer, 2018). Standards can be developed for ideal public data sets and then leveraged to achieve optimal states of individual health, striking a balance between research and implementation (Özdemir and Springer, 2018). Addressing the larger social determinants of health and allowing the health system to actively meet the health needs of people continuously require adequate resource mobilization, which AI/ML can help to accomplish. Employing AI/ML to orchestrate the supply chain, allocate resources, reduce counterfeit and falsified drugs, and predict demand can potentially eliminate unforeseen stock outs and improve overall access to health care needs (Stauffer and Buckley, 2005).

As the vision of PHC calls to undo the reactive, episodic nature of health care, AI/ML for health should follow in suit and match this PHC plan. Many global actors in the Digital Health and ICT fields have been able to pull together and develop guidance and principles to align use of AI/ML to achieve the SDGs and UHC (Druetz, 2018; The IEEE Global Initiative on Ethics of Autonomous Intelligent Systems, 2019; Truong et al., 2019; Flahault et al., 2020). Yet even if developed according to best practice, all implementations of AI/ML in health are still dependent on the current parameters of the health system into which they are deployed. Therefore, if the health system is still reactive and has not shifted toward prevention, AI/ML will be limited as a reactive weapon, unaligned with the components of a PHC future.

Ultimately, the greatest challenge to enlisting AI/ML in the quest toward the PHC vision will be ensuring that it also shares the human-centered focus in all efforts. This challenge is not representative of technology capabilities, but of our own society's mindset consisting of knowledge, attitude, and behavioral intentions toward AI/ML as a tool for preventative PHC. Consequently, achieving this change in mindset relies upon a series of actions required to prioritize PHC and prevention due to the understanding of the capabilities of what health data and AI/ML can do (Table 1).

**Table 1:** Recommendations to shift the collective mindset toward using AI/ML for PHC.

1	Place primary focus on increasing the general knowledge of AI/ML and educate at all different levels, particularly within LMICs; build more human capacity to work with AI/ML, including more diverse people in the assessment and evaluation of AI/ML.
2	Build trust between AI/ML, health care providers, and patients through explanations of AI/ML; commit to implementable ethical frameworks (World Health Organization, 2018, 2019; Wiegand et al., 2019).
3	Set and incentivize positive behavioral intentions behind AI/ML usage for health.
4	Define the value exchange of health data between the individual and public for the greater global good.

Education and increased awareness of AI/ML are key components to this social and behavior change, but we need to make sure AI/ML is also “transparent” or “explainable” for people to begin to understand how it can augment health care (Truong et al., 2019). Even more so in the health field, the explanation of a model or a prediction determines a medical provider's trust in using AI/ML to augment their practice (U.S. Agency for International Development (USAID), 2019). Common understanding of the reasoning behind predictions or providing explanations of a model will build trust between a health provider using AI/ML for the patient and the patient using AI/ML for their own empowerment and care (U.S. Agency for International Development (USAID), 2019). Various techniques are now being developed and used to improve the explanations of AI/ML models and predictions, such as explainable feature engineering, adoption of explainable model families, and proxy models (U.S. Agency for International Development (USAID), 2018). Trust of models and predictions should be questioned by not just anyone but by a diversified and contextualized set of implementors with relevance to the outcomes of that particular model or prediction (UN Secretary-General's Independent Expert Advisory Group: Data Revolution Report, 2014). Evaluation of trust of models and predictions by end users, local stakeholders, and a varied demographic is a strategic approach to implementing unbiased models and an imperative standard we must keep for all health systems. Trust also needs to be built by data rights upholding human rights; data rights for global citizens will need to be fully realized, including standards for anonymizing data that are personally identifiable and global standards and enforcement mechanisms for data security, integrity, documentation, preservation, and access (Watts, 2019).

When this trust is built with society and perceived individual risk is mitigated, then the necessary confidence will endure for people to consent to use of their data toward the global good (Cardona-Salazar et al., 2019; Watts, 2019). Therefore, a value exchange of health data between the individual and public must be defined; what information are people willing to give away for the global good, in return for what individual gain? A truly human-centered approach to leveraging AI/ML for health is when individuals understand, trust, and appreciate the value gained from a health data exchange. That is when society's mindset of health care will have actually shifted, allowing utilization of AI/ML for the goal of prevention and realization of PHC. Hence, especially in LMICs, deficient AI/ML education and awareness, lack of trust in the health system, and a non-existent value for health data all represent a mindset that sticks out as the most glaring challenge and one that will be harder to overcome than the acceleration of AI/ML technologies and its widespread use within flawed health systems.

The technological capabilities of AI/ML are at our fingertips today, and resolutions to the biggest problems in data science are quickly approaching with explanations of opaque models and implementation of models with real-world data. The greatest challenge will soon only be our collective mindset missing the knowledge, attitude, and behavioral intentions required to using AI/ML for PHC in LMIC health systems. To resolve this, action is needed now to fill these gaps and emphasize value clarification toward the PHC vision, unlocking the global good that can come from utilizing AI/ML.

## Author Contributions

ET designed, wrote, and edited the entire manuscript.

## Conflict of Interest

The author declares that the research was conducted in the absence of any commercial or financial relationships that could be construed as a potential conflict of interest.
